# Mixture Effects of Commonly Applied Herbicides on County Level Obesity Rates in the United States: An Exploratory Ecologic Study (2013–2018)

**DOI:** 10.3390/toxics13100894

**Published:** 2025-10-19

**Authors:** Sarah Otaru, Laura E. Jones, David O. Carpenter

**Affiliations:** 1 Department of Environmental Health Sciences, College of Integrated Health Sciences, State University of New York at Albany, 1400 Washington Avenue, Albany, NY 12222, USA; dcarpenter@albany.edu; 2 Center for Biostatistics, Bassett Research Institute, 1 Atwell Rd, Cooperstown, NY 13326, USA

**Keywords:** mixture models, glyphosate, herbicides, obesity rates, rurality, NCHS urban–rural designation

## Abstract

Metabolic disorders such as obesity have increased globally in recent decades and are a major public health concern. Previous research suggests that herbicide exposures may contribute to metabolic dysfunction, but few studies have examined mixture effects of multiple herbicides on obesity at a population level. Using county-level data from 2013 to 2018, we examined the associations between obesity rates and the application of 13 commonly applied herbicides in the U.S. We first conducted adjusted single-pollutant mixed-effects models and then used quantile-based g-computation mixture modeling to assess combined herbicide mixture effects on county-level obesity rates. Models were adjusted for demographic and socioeconomic covariates and accounted for geographic clustering. Significant positive associations were identified between county-level obesity rates and applications of glyphosate, 2,4-D, atrazine, acetochlor, metolachlor, and several other herbicides in adjusted single-pollutant models. Glyphosate showed one of the strongest individual associations (β = 0.29 per standard deviation increase, 95% CI: 0.21–0.36). Increases in herbicide mixture were significantly associated with higher obesity rates (Psi = 0.71 per quantile exposure mixture, 95% CI: 0.65–0.76) from mixture modeling. Inclusion of significant interaction terms did not appreciably increase the mixture effect. Glyphosate, 2,4-D, metolachlor, dimethenamid-P, and glufosinate contributed most strongly to the weighted mixture effect. Mixture effects varied by rurality, with stronger associations observed in rural counties, particularly in micropolitan regions. Our findings highlight the importance of considering cumulative herbicide mixture exposures rather than individual chemicals in isolation. The observed rural–urban disparities emphasize the need for targeted public health interventions and policy actions in rural communities, which may be particularly vulnerable to the adverse metabolic impacts of herbicide mixtures.

## 1. Introduction

Recent decades have seen a substantial increase in human metabolic disorders—including obesity, diabetes, and metabolic syndrome—which now represent a major public health challenge in the United States and globally [1,2]. Agricultural practices have evolved in tandem with the widespread application of pesticides. Of these, herbicides are by far the most heavily applied class of pesticides, accounting for ~88% of agricultural herbicide mass during 2013–2017 in the United States, whereas insecticides and fungicides each contributed ~6%. Moreover, by 2016 glyphosate alone comprised ~44% of all herbicide mass applied to crops [3,4]. Our prior study demonstrated a significant association between glyphosate exposure, measured in urine, and metabolic syndrome using nationally representative NHANES data, with marked differences observed across racial groups [5]. These findings suggest that herbicide exposure may be an underappreciated environmental co-factor of metabolic dysfunction. However, given that agricultural herbicide exposures rarely occur in isolation, a comprehensive analysis that includes multiple compounds is warranted to elucidate interactive and mixture effects on metabolic health.

In the present study, we extend our investigation with an ecologic study of herbicide application and its associations with county-level obesity rates by incorporating a panel of thirteen herbicides—glyphosate, herbicide 2,4-D (2,4-D), atrazine, dicamba, trifluralin, acetochlor, dimethenamid-P, glufosinate, metolachlor, metolachlor + metolachlor-S, pendimethalin, paraquat—and one fungicide—chlorothalonil—that are most extensively applied in U.S. agriculture and have been the subject of extensive toxicologic and epidemiologic evaluation due to their widespread application and potential health and environmental impacts [6]. In addition glyphosate applications have increased dramatically since 1996 (15-fold by 2016) following the development of “Roundup^TM^-ready” crops [7]. Enthusiastic adoption of “Roundup^TM^-ready” crops has resulted in increased applications of dicamba and herbicide 2-4D to manage herbicide-resistant weeds. The selection of the fourteen compounds studied here was informed by multiple criteria: (1) their high prevalence in agricultural use in the United States, and documented potential for environmental persistence; (2) emerging toxicological evidence implicating them in endocrine disruption, oxidative stress, and perturbations in glucose and lipid metabolism [8,9,10]; and (3) epidemiological observations that link these exposures to adverse metabolic outcomes [11]. Importantly, many of these herbicides are applied together in modern cropping systems, potentially leading to synergistic and mixture effects that are not adequately captured by single-pollutant studies.

The biology linking these compounds to metabolic disturbances is supported by several mechanistic pathways. For instance, glyphosate has been implicated in disrupted insulin signaling and altering the gut microbiome—both of which are critical regulators of energy homeostasis and metabolic function [12,13,14,15]. Similarly, atrazine and 2,4-D have been associated with endocrine disruption, which may interfere with hormonal regulation of adipogenesis and glucose metabolism [10,16]. The neurotoxic potential of paraquat [17,18] further raises concerns about systemic oxidative stress, a contributor to insulin resistance and beta-cell dysfunction [19,20]. In integrating diverse lines of evidence, our study seeks to clarify whether cumulative exposures to herbicides contribute to metabolic disorders at a population level.

After assessing associations between county-level obesity rates and single pollutants using mixed-effects models with a random term for county FIPS, we use a mixture model approach to assess mixture effects, using a county-level random effect to account for location. This approach enables us to accommodate geographic variations in herbicide usage and to control for spatially specific local demographic and socioeconomic factors that may modify exposure–outcome associations. Finally, by stratifying analyses on National Center for Health Statistics (NCHS) urban–rural designation, we aim to reveal potential environmental health disparities that could inform targeted public health interventions.

**Mixture effects.** Mixture effects occur when multiple environmental pollutants interact in ways that amplify their individual health effects, and may be more common when compounds are in related families [21,22]. Pollutant mixtures may have joint effects [23], even at low doses [24,25], and if there are interactions between components the total mixture effect can be amplified [26]. As pollutant mixture effects are a long-recognized source of health risk, methods to assess the effects of pollutant mixtures, primarily for air pollutant mixtures, were in use as early as 2000 [27]. Despite some focus on acute herbicide exposure (poisoning), the effects of chronic ambient environmental exposure herbicide mixtures are little studied in view of increasing global application of herbicide mixtures for everything from weed control in cropping to crop desiccation, aquatic or wildland weed control, and urban area management [28,29].

In summary, our study addresses critical gaps in the current understanding of environmental risk factors for metabolic disorders. By examining a spectrum of herbicides with documented endocrine and metabolic perturbations, this study provides a nuanced assessment of the complex interplay between agricultural chemical exposures and metabolic health outcomes as a function of exposure setting and magnitude. Such insights are vital for informing regulatory policies and for designing future studies that can elucidate the causal pathways underlying these associations.

## 2. Materials and Methods

### 2.1. Study Design

Our study uses aggregated county-level exposure and outcome data to explore relationships between herbicide exposure and health outcomes. Because both exposure and outcome comprise county-level measurements, there is an inherent study assumption that agricultural herbicide applications are correlated with local population exposure. This is biologically and empirically plausible because urinary glyphosate concentrations rise during the agricultural spray season among residents living near fields [30,31], indicating that local application intensity tracks human exposure. Diet may contribute, but provides a smaller contribution to exposure for subjects living in proximity [32]. Moreover, our prior NHANES study demonstrated an association between urinary glyphosate measurements and metabolic syndrome score, increasing confidence that population-level exposure can serve as a meaningful proxy for human exposure in this context [5]. The U.S. Geological Survey (USGS) [33] provides estimates of agricultural pesticide and herbicide application in kilograms, aggregated by FIPS code and year, which we employ alongside county-level obesity rates from the Behavioral Risk Factor Surveillance System (BRFSS) and demographic information from the American Community Survey (ACS) for study years 2013 to 2018. Sources are described further below.

### 2.2. Data Sources, Curation, and Integration

We curated a county–year analytic file by integrating multiple public, nationally representative sources and joining records by five-digit FIPS and calendar year. Agricultural herbicide use estimates were obtained from the U.S. Geological Survey (USGS) Pesticide National Synthesis Project (e-Pest), for which peer-reviewed evaluations of data generation and occurrence are available [3,4]. County-level adult obesity prevalence was drawn from CDC small-area estimation based on BRFSS (PLACES), which uses multilevel regression and post-stratification and has been validated for local health indicators [25,26]. Socio-demographic and behavioral covariates (e.g., smoking, uninsured, unemployment, education, age structure, race/ethnicity) were curated from the University of Wisconsin’s County Health Rankings, which aggregate the American Community Survey and other federal series under a documented framework [34,35]. This approach mirrors recent ecologic studies that explicitly curate and integrate PLACES and other public datasets for population-level analyses [36,37,38].

### 2.3. Designation of Rurality

Rural–urban setting designations are from the 2013 National Center for Health Statistics (NCHS) Urban-Rural Classification Scheme for counties and include six categories: “large central metro”, “large fringe metro”, “medium metro”, “small metro”, “micropolis” and “non-core.” Details for each category are shown in Table 1. The 2013 NCHS scheme is based on the 2010 census and the February 2013 Office of Management and Budget (OMB) delineation of metropolitan statistical areas (MSAs) and micropolitan statistical areas, where a micropolitan statistical area is defined as a population of 10,000 to 49,999. There were 374 metropolitan statistical areas and 581 micropolitan statistical areas defined as of 2013. These designations were recently updated to a 2023 standard using 2022 census data, but our data comprise years 2013 to 2018; thus, we employ the 2013 system. Note that there were minimal changes to county assignments between 2013 and 2023.

### 2.4. Exposure

Primary exposure variables include the estimated annual agricultural use of atrazine, dicamba, trifluralin, acetochlor, chlorothalonil, dimethenamid-P, glufosinate, glyphosate, herbicide 2,4-D, metolachlor, metolachlor-S, metolachlor + metolachlor-S, pendimethalin, and paraquat. Herbicides were selected based on application levels across U.S. counties as follows: Exposure estimates were downloaded from the USGS e-pest (Pesticide National Synthesis Project [33]) site for years 2013 through 2018. E-pest estimates are reported as “low” and “high” values (in kilograms) for 448 unique compounds over the time period for most states. California supplies only one comprehensive number per county, taking data directly from its comprehensive Pesticide Use Reporting (PUR) database. For the remaining states, we computed averages of low and high values (in kilograms) for each exposure, then omitted exposures with a total mean value of less than 1400 kg/hectare, irrespective of missing units, resulting in 43 candidate exposures. Columns with more than 30% missing units were dropped, resulting in the final 14 exposures listed above. Remaining missingness varied from <1% to at most 20%. State and county FIPS numbers were joined to create five-digit FIPS values for each county, and exposures were merged with county-level outcome and demographic data based on study year and county FIPS number.

### 2.5. Outcome

Adult (ages 18 and older) obesity data are compiled by the CDC from the Behavioral Risk Factor Surveillance System (BRFSS). The BRFSS is an annual state-level random digit dial survey used to assess health and risk-related behaviors. From 2016 onwards, the CDC employed a multilevel modeling approach to estimate obesity, along with other health conditions, based on telephone survey responses and respondent age, sex, and race/ethnicity, combined with county-level poverty and other relevant county- and state-level features [39,40]. For counties where there is insufficient data, the approach borrows data from the entire BRFSS sample as well as old census estimates, using a parametric bootstrap to produce standard errors. A companion study will examine associations between county-level obesity, hypertension and hypercholesterolemia rates (%), and herbicide exposures within larger Metropolitan Statistical Areas (MSAs), using data from CDC Places for 2013 to 2018. The CDC uses a similar multilevel modeling approach to estimate CDC Places health outcomes.

### 2.6. Covariates

Additional demographic and socioeconomic variables—including adult smoking prevalence, percent uninsured, percent unemployed, age structure (≥65 and <18 years), educational attainment (percent high school graduate; percent with some college), racial/ethnic composition, and county rurality (NCHS 2013)—were included a priori to address confounding. Percent uninsured comprises age-adjusted prevalence of subjects aged 18 to 64 who lack health insurance; percent smoking reflects the percentage of the county population who currently smoke and have smoked more than 100 cigarettes in their lifetime. Smoking was treated as a key confounder because rural smoking prevalence exceeds urban prevalence, including in New York State and even after accounting for poverty, and because smoking is associated with adiposity/metabolic risk behaviors. To partially address dietary confounding—for which no county-level intake data were available—we included food insecurity and educational attainment as proxies for diet quality/access and health behaviors; recent national evidence shows poorer diet quality in non-metropolitan/rural areas independent of income/education and food-desert status [41]. These county-level metrics are compiled by study year and made available for download by the University of Wisconsin Population Health Institute (https://www.countyhealthrankings.org, accessed on 24 September 2024) and are also available at the Centers for Disease Control.

### 2.7. Statistical Analysis

Due to the distributed nature of missing units across county-level herbicide estimates, missingness decreases our sample size from 18,382 samples to 7811 samples if we take a complete case analysis approach. To preserve sample size, data were thus first multiply imputed using a fully conditional chained equations approach implemented in R via the mice package, creating 10 imputed datasets, each given 20 iterations to allow convergence, and using a custom prediction matrix. Please see Appendix A for details of the imputation process and model.

### 2.8. Single Pollutant Models

**Figure A1:**
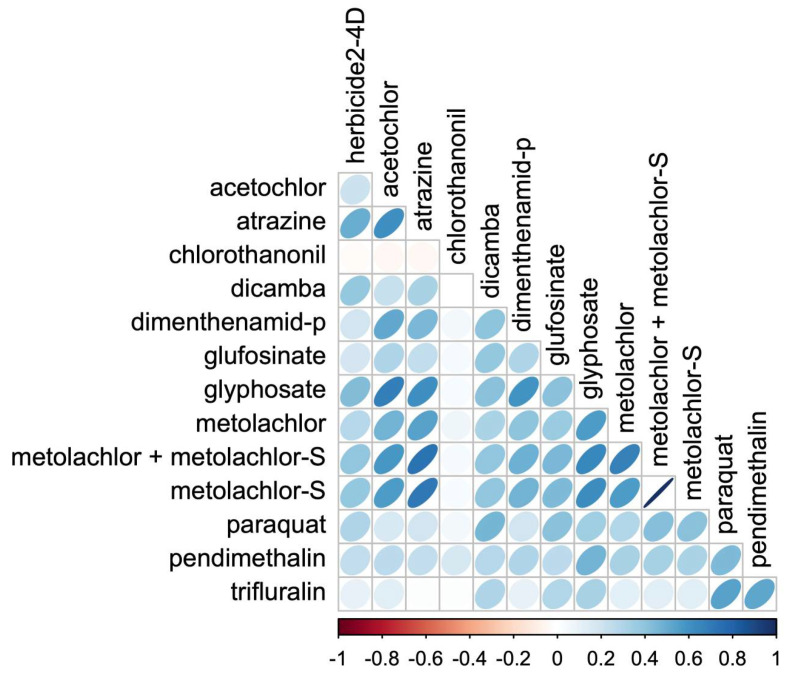
Pearson correlations of all county-level imputed herbicide exposures from NEI data.

After imputation and before multivariable analysis, we assessed potential collinearity between herbicide application data by computing and visualizing a Pearson correlogram (Appendix A, Figure A1), as multicollinearity will inform further modeling. We then examined single-pollutant associations using mixed-effects models adjusted for racial percentages, percentage age 65 and above, and percentage below age 18; county-level smoking rates; percentage uninsured, unemployed, and food-insecure; and NCHS rurality designation, with a random effect to account for county-level variability. Exposures were mean-standardized before analysis and the results are reported as per standard deviation exposure, and results were pooled over 10 imputed datasets using Rubin’s Rules [42]. *p*-values were adjusted for multiple comparisons via the False Discovery Rate (FDR) method [43]. Results are given in tabular format and visualized as forest plots. A second set of adjusted single-pollutant regressions with the exposure categorized by quintile was run to assess potential for nonlinear dose–response relationship.

### 2.9. Mixture Models

Our data showed correlation between the herbicides; thus, a standard multivariable model was inappropriate due to potential multicollinearity and associated variance inflation, unstable coefficient estimates, and difficulty assessing exposure importance. Correlation analysis warns us of potential multicollinearity in our models yet cannot detect which correlated exposures are driving the associations, or whether there are significant interactions between the pollutants; we rely upon other methods to assess this. As the size of our dataset makes application of flexible nonparametric kernel-based methods such as Bayesian Kernel Machine Regression (BKMR) [44,45] computationally impractical, we used quantile-based g-computation models to estimate the joint effects of herbicide applications at the county level on county-level obesity rates [46]. Based on our correlation analysis (see Appendix A, Figure A1), we dropped Metolachlor-S from the mixture components. We first ran an unadjusted model to examine crude mixture effects, and then a model adjusted for racial percentages, percentage age 65 and above, and percentage below age 18; county-level smoking rates; and percentages uninsured, unemployed, and food-insecure, using a grouping term to account for clustering at the county level and a bootstrap approach to correctly estimate variances. Optimal quantile settings were selected by examining Z-scores and were set at 15 quantiles for both crude and adjusted models. Quantile-based g-computation models were run using the gcomp package in the R programming language [46].

### 2.10. Interactions

Potential interactions between herbicide exposures were assessed for inclusion in g-computation models by running interaction forests, a variant of the random forest (RF) method that explicitly captures interaction effects in the bivariable splits performed by the decision trees in RF, using the diversity Forest package in R [47].

### 2.11. Stratified Mixture Models/Subgroups Analysis

Since application of herbicides in farming is associated with rurality, we perform mixture modeling stratified on NCHS Urban-Rural designation.

## 3. Results

### 3.1. Study Population

The study population resides in 3066 counties spread across the United States (Table 2). Only 13.5% of the counties are in large metropolitan or large fringe metropolitan areas. In total, 63% of counties are in either micropolitan or non-core areas. Our study population is largely (median 86.3) non-Hispanic White and has graduated high school (86%). Over half have attended college (56%). County-level smoking rates are about 19%, most people are employed, and only 16% are without health insurance, with only 14% food insecurity. County-level obesity rates across the study period have mean and median values of 31% and rates are normally distributed.

### 3.2. Herbicide Exposure and Health Outcomes by Rurality

Herbicides of interest in this study had median annual application levels ranging from 128 kg per hectare (chlorothanonil) to 15,360 kg per hectare (glyphosate). Maximum annual applications ranged from 70,608 kg (metolachlor) to 594,336 (glyphosate) kg per hectare (Appendix A, Table A1). Annual applications at the county level generally increased with increasing rurality, flattening out as rurality increased above 45%. Counties within micropolitan urban–rural designations (52.3% median rurality) had the highest median annual applications of metolachlor species, herbicide 2-4D, atrazine, acetochlor, dimenthenamid-P, and glufosinate, and the second highest applications of glyphosate (Appendix A, Table A2). Large metropolitan areas and metropolitan fringe areas had the lowest application levels. Mean/median obesity rates also increase with rurality, again flattening out at about 31% at rurality 45% and above (NCHS small metro, micropolitan and non-core regions).

### 3.3. Single Pollutant Models

**Figure 1:**
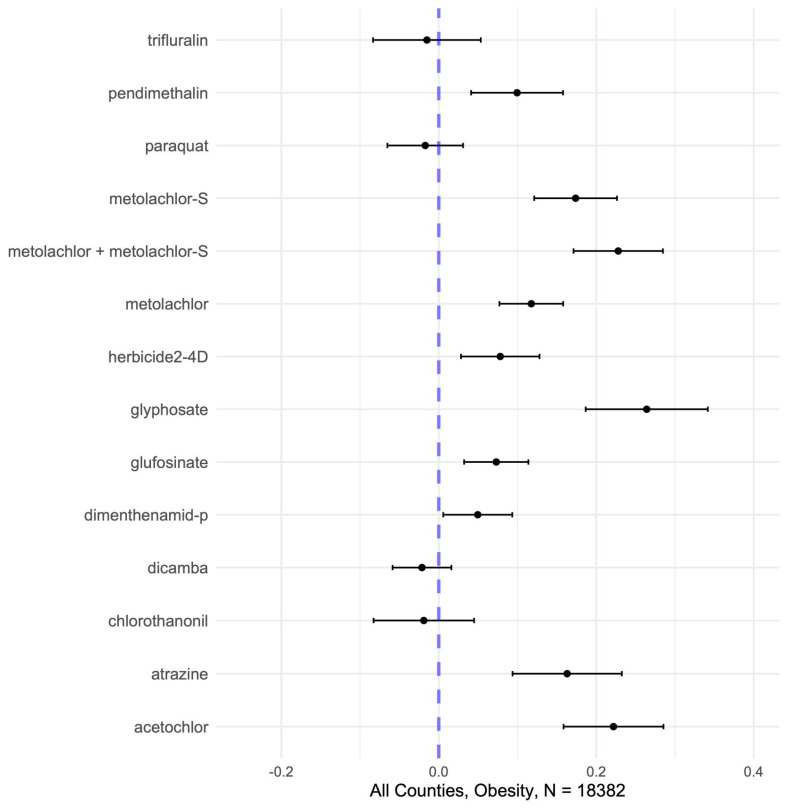
Forest plot of single-pollutant results for associations between county-level obesity rates and herbicide applications from adjusted mixed-effects models, years 2013 to 2018. Results pooled over 10 imputed datasets.

Across all counties, the adjusted analysis revealed positive single-pollutant associations between glyphosate, metolachlor and metolachlor-S, acetochlor, atrazine, pendimethalin, herbicide 2-4D, Glufosinate, and Dimenthenamid-P applications (in decreasing magnitude), respectively, and county-level obesity rates (Figure 1, Table 3). Associations for glyphosate were strongest (+0.30 per SD exposure, 95% CI: 0.2, 0.4), followed by metolachlor + metolachlor-S (+0.24, 95%CI: 0.2, 0.3) and eight other compounds. A total of 10 of 14 county-level herbicide applications showed positive and significant associations with obesity rates, though those for dimenthenamid-P were marginal after correction for multiple comparisons. Trifluralin, paraquat, dicamba, and chlorothanonil applications show no significant adjusted linear associations with county-level obesity rates. Quantile regressions with exposure categorized by quintile show little evidence of a nonlinear dose–response relationship in most herbicides. Results categorized by quintile show that 13 of 14 herbicides are significantly positive and monotonically increasing, and all are significant for at least two of the four reported quintiles (Appendix A, Table A2). Again, the largest positive associations are shown for glyphosate, followed by metolachlor + metolachlor-S and 11 other herbicides.

### 3.4. Mixture Models

**Figure 2:**
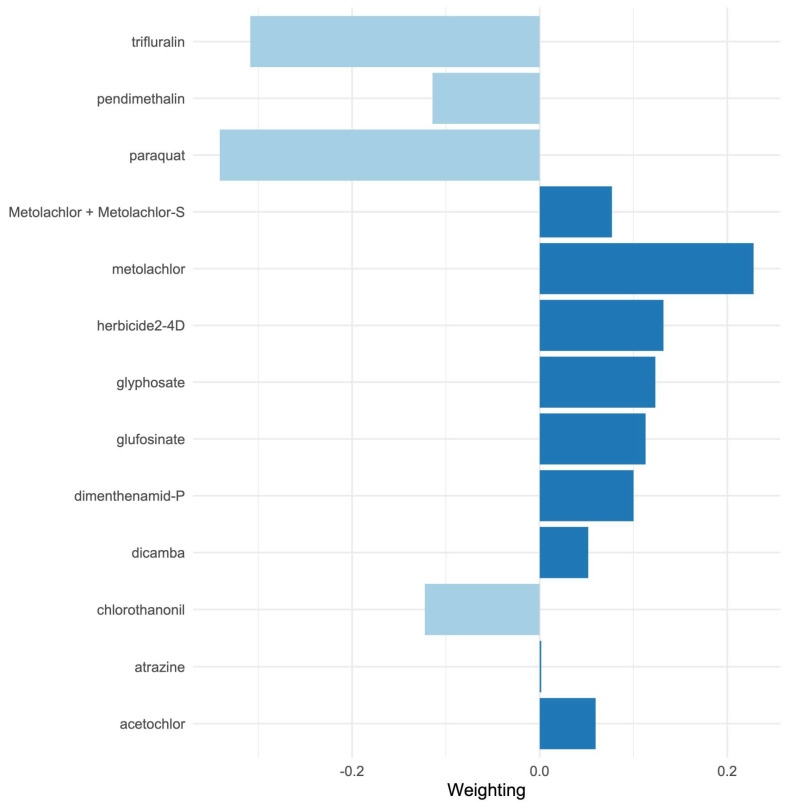
Mixture weightings from the final adjusted (unreduced) quantile g-computation mixture models for county-level obesity rates. Negative weights are shown in pale blue and positive weights in dark blue. Results are pooled over 10 imputed datasets. Metolachlor-S is omitted from the mixture models due to very high correlation with other metolachlor species (see Appendix A, Figure A1). For a coefficient (beta estimate) forest plot from the final fitted mixture model, please see Appendix A, Figure A2.

**Figure A2:**
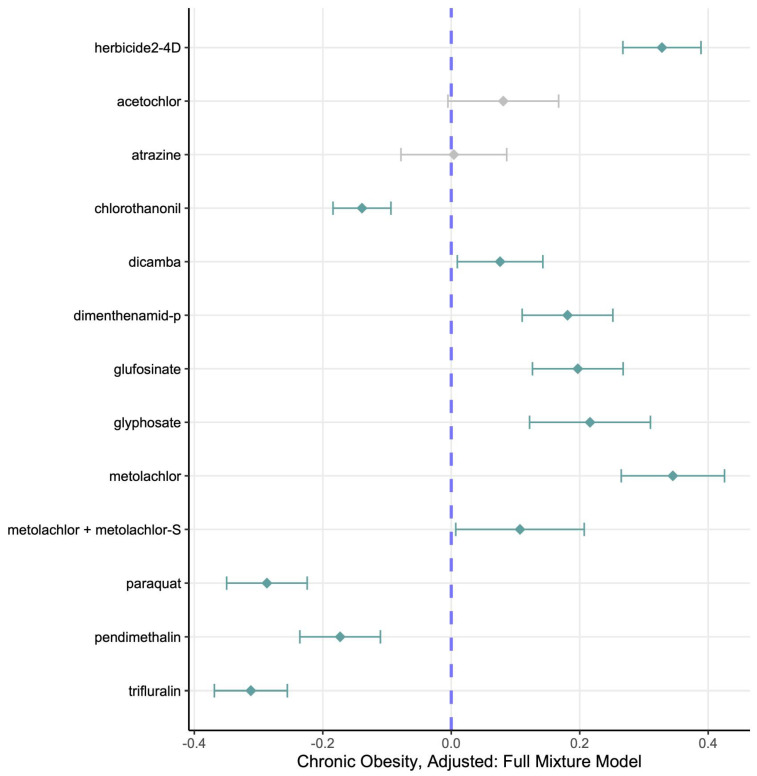
Coefficient (beta) estimates extracted from the final unreduced, adjusted, bootstrapped quantile g-computation mixture models (multi-pollutant) for associations between county-level obesity rates and a mixture of herbicides from adjusted standardized models. Models are adjusted for percent smoking, percent uninsured, percent unemployed, education level, race, age (percent under 18 or over 64), study year, and NCHS rurality designation. County is included as a grouping ID.

**Figure A3:**
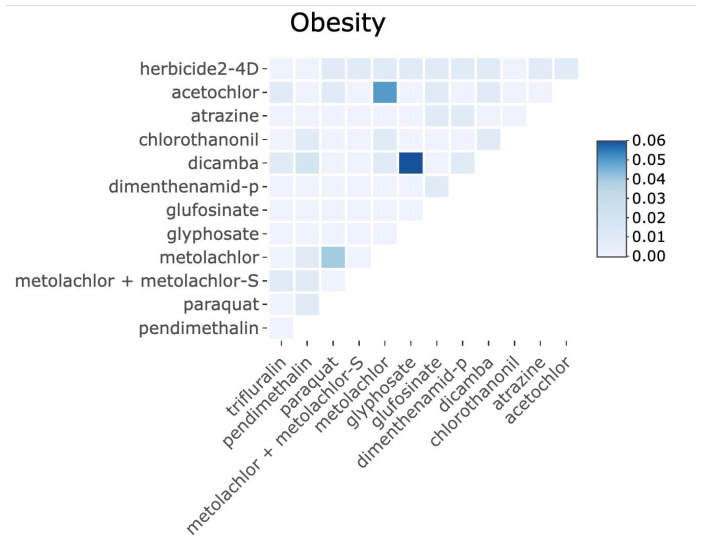
EIM strengths of pairwise quantitative (direct) interactions for mixture components and obesity rates from interaction forests including all environmental exposures and covariates. There are no qualitive (cross-over) interactions.

In unadjusted models, a one-quantile increase in herbicide application mixture was associated with a significant increase in obesity rates (ψ = 1.02, 95% CI: 0.94, 1.1; *p* < 0.0001, Table 4). Adjusted models showed slightly reduced but still significant mixture effects with a one-quantile increase in obesity rates (ψ = 0.71, 95%CI: 0.65, 0.76, *p* < 0.0001). Model weightings for the mixture effects showed large positive contributions from herbicide 2-4D, metolachlor, glyphosate, dimenthenamid-P, and glufosinate, and large negative contributions from paraquat, trifluralin, and pendimethalin (Figure 2). Here, positive weights indicate positive additive effects on obesity rate, whereas negative weights are negative contributions for a given component in the per-quantile mixture effect. For a forest plot of coefficients (beta values) from the underlying fitted mixture model, please see Appendix A, Figure A2. Surprisingly, the interactions assessed via interaction forests are few. The largest, a quantitative interaction between dicamba and glyphosate, had an Effect Importance Measure (EIM) strength of 0.06 (Appendix A, Figure A3). Adjusted mixture models incorporating these interactions, however, do not produce significantly different total mixture effects (Table 4).

### 3.5. Mixture Analysis Stratified by Rurality

**Figure 3:**
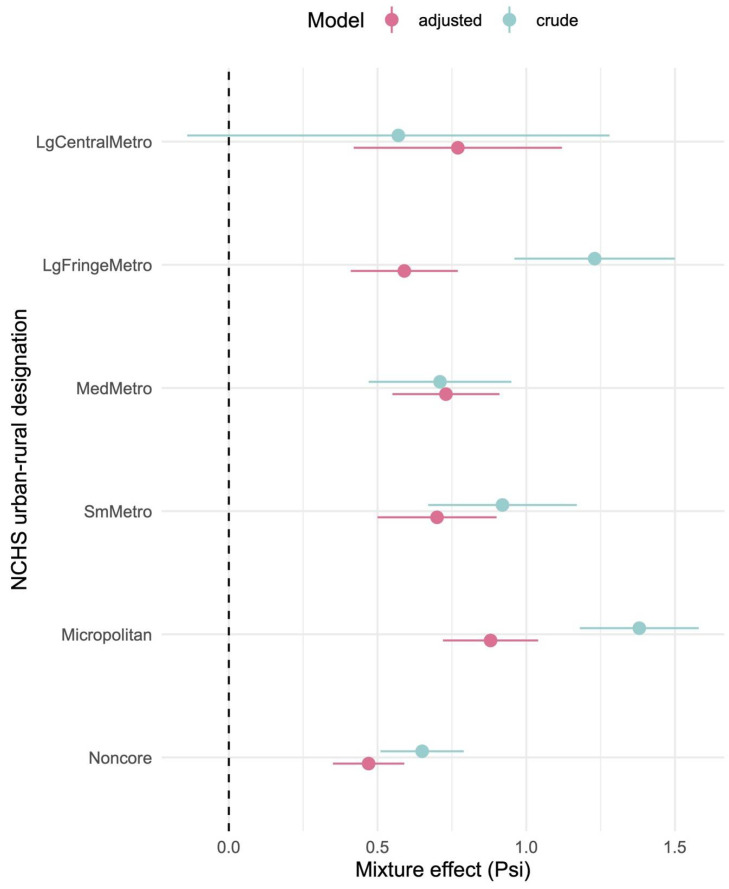
Forest plot of mixture effects (Psi) from subgroup analysis via mixture models stratified on NCHS urban–rural designation, presented with confidence intervals. Adjusted models are shown in dark rose, and mixture-only (crude) models are shown in pale blue.

**Figure 4:**
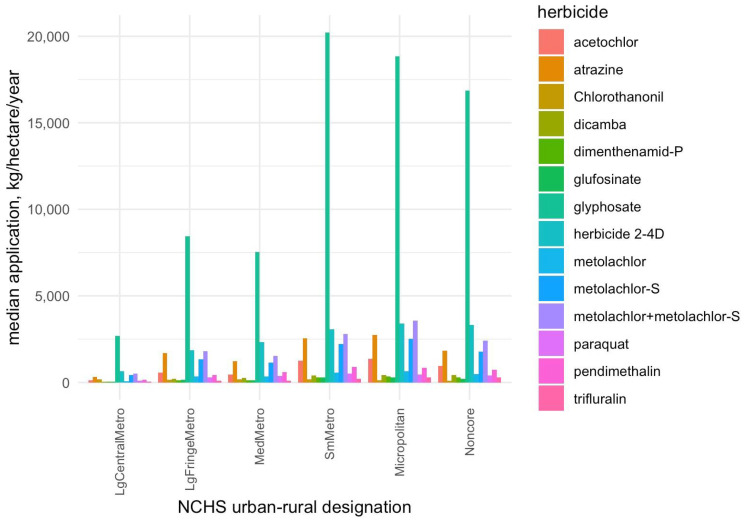
Median herbicide application by NCHS urban–rural designation (kg/hectare/year). See also Appendix A, Table A4.

As in unstratified models, mixture-only models (estimates and confidence intervals are shown in pale blue in Figure 3) generally had higher mixture effects per quantile exposure mixture than adjusted models. Large central metros are an exception, but the sample size is also much smaller (comprising fewer counties) than other levels. Mixture effects from adjusted models rise with increasing rurality from large fringe metropolitan (Psi (ψ) = 0.6, 95%CI: 0.4, 0.8) to micropolitan areas (Psi (ψ) = 0.9, 95%CI: 0.7, 1.0, see Appendix A, Table A3). Surprisingly, non-core regions have among the lowest mixture effects, though the intercepts for this area show the highest rates of baseline obesity (Table A4). Note however that median exposures for most herbicides peak in small metropolitan and micropolitan districts during the period of interest (see Figure 4 and Appendix A, Table A4).

## 4. Discussion

Using freely available public data on county-level obesity and application of the 14 most commonly used herbicides in a five-year period (2013–2018), we have found significant adjusted associations between county-level obesity rates, individual herbicides, and their mixture. We have shown that these associations roughly mirror pesticide applications, and that these are in part, but not fully, associated with rurality and setting.

Our analysis demonstrates that U.S. counties with greater herbicide use, including glyphosate and several other common agents, tend to exhibit higher obesity rates, consistent with prior evidence that herbicide exposure contributes to adverse metabolic outcomes [48,49]. We observed significant positive associations from single-pollutant models between county-level obesity prevalence and the application of multiple herbicides (10 of the 14 analyzed in standardized continuous data, 12 of 14 in quantile categorized), most notably glyphosate, acetochlor, metolachlor -S, metolachlor (including its S-isomer), atrazine, metolachlor, pendimethalin, 2,4-D, glufosinate, and dimenthenamid-P (Figure 1), as well as some evidence of a nonlinear dose–response relationship. Glyphosate, as the most heavily used herbicide (Figure 4), showed the strongest individual associations with obesity, followed by 2,4-D and atrazine, consistent with human studies linking these herbicides to metabolic syndrome, of which obesity is a key component [5,50,51,52,53,54]. Experimental studies provide plausible biological mechanisms by which these herbicides may influence metabolic outcomes like obesity. Glyphosate has been shown to alter gut microbiota composition, increase oxidative stress, and up-regulate expression of the NF-κB (Nuclear Factor kappa-light-chain-enhancer of activated B cells), all of which can impair insulin signaling and promote adipogenesis [14,55,56]. Atrazine and 2,4-D have been linked to endocrine disruption through interference with estrogenic and thyroid pathways, activation of PPARγ—a transcription factor central to fat storage and adipocyte differentiation—and mitochondrial dysfunction, leading to insulin resistance [50,57]. These mechanistic insights support the plausibility that chronic exposure to herbicide mixtures could dysregulate energy balance, lipid metabolism, and glucose homeostasis, thereby contributing to obesity risk.

Not all compounds had significant linear independent effects in continuous data (e.g., dicamba, paraquat, trifluralin, and chlorothalonil showed no positive single-pollutant association with obesity in continuous data). However, most showed partial positive effects (usually the highest levels, Q4 and Q5 relative to Q1) in quintile categorized data, highlighting that the health impacts of herbicides are not uniform across compounds, and that slight nonlinearities may mask significant associations when examined in continuous data. This heterogeneity likely reflects different toxicological mechanisms and exposure patterns which may influence metabolic outcomes in subtle and indirect ways.

Importantly, our mixture modeling analysis suggests that combined exposures to herbicides may better explain population obesity patterns than individual chemicals alone. Using quantile-based g-computation, we found that a one-quantile increase in the overall herbicide mixture was associated with a significant increase in county obesity rates, even after adjusting for socioeconomic covariates. In the adjusted mixture model, herbicides such as 2,4-D, glyphosate, metolachlor, glyphosate, dimethenamid-P, glufosinate, metolachlor (including its S-isomer), dicamba, acetochlor, and atrazine showed the largest positive weight contributions to this mixture effect, whereas others (paraquat, trifluralin, pendimethalin, and chlorothalonil) contributed negative weights. Notably, some herbicides that showed null or modest single-pollutant associations (e.g., paraquat) exhibited negative contributions in the mixture context, possibly reflecting complex interactions (see Appendix A, Figure A3) or spatial usage patterns that are not fully accounted for in the mixture model (for instance, areas with high paraquat use may have lower use of other obesogenic herbicides, diluting the overall mixture effect). On the other hand, glyphosate—while strongly associated on its own—did not singularly dominate the mixture effect, as several co-occurring herbicides also exerted substantial influence. These results underscore that glyphosate is not uniquely responsible for the observed obesity link; rather, it is one significant part of a total herbicide mixture effect. In practical terms, evaluating herbicides in isolation may bias risk estimates, whereas accounting for concurrent exposure mixtures provides a more realistic assessment of environmental influences on obesity. Our study is among the first ecological analyses to examine herbicide mixtures in relation to metabolic health, and it provides novel evidence that the cumulative effects of multiple agrichemicals are relevant to population obesity rates.

### 4.1. Contextualizing Rural–Urban Disparities

A notable finding from our study is the heightened impact of herbicide exposure in more rural areas. herbicide usage tends to be highest in counties of intermediate-to-high rurality (e.g., small metropolitan and micropolitan areas) and lowest in major urban centers (Figure 4). Consistently, we observed that the overall herbicide mixture effect on obesity was more pronounced in more rural counties: the estimated obesity increase per mixture quartile was smallest in large metropolitan areas and grew larger moving toward small metro and micropolitan counties. Interestingly, mixture effects in the most remote rural counties (“non-core” areas) did not follow a strictly linear trend—they showed the highest baseline obesity rates but a somewhat lower mixture effect estimate—suggesting that beyond a certain point, additional exposures may yield diminishing returns, or that these communities may face saturated obesity risk from many non-measured contributors. Overall, however, the pattern indicates that communities with intensive agriculture (often rural) may experience a double burden: greater chemical exposure alongside underlying vulnerabilities such as higher poverty, limited health care access, and other lifestyle risk factors [58]. These contextual factors can amplify the health impact of environmental exposures. Indeed, rural populations often have fewer resources to mitigate or treat chronic conditions, which could exacerbate the observed associations. Our findings therefore support calls for region-specific public health interventions and regulatory approaches. In practice, this could mean prioritizing cumulative risk assessments for heavily agricultural rural regions and tailoring obesity prevention programs to address both lifestyle and environmental factors in these communities. By recognizing that rurality can be a proxy for both elevated exposure and increased susceptibility, public health officials can better target efforts to reduce herbicide exposure and bolster health care support in high-risk counties. Recent work has underscored how socioeconomic and infrastructural disparities can modify the health impacts of environmental exposures [58].

### 4.2. Confounding by Smoking and Diet

We adjusted for county-level adult smoking prevalence because rural smoking remains higher than urban smoking in the U.S. after adjustment for socio-demographics, showing substantially higher smoking in rural/upstate counties even where poverty is high [59,60]. We adjusted for diet proxies, specifically food insecurity and educational attainment, because both are well-established county-level predictors of diet quality, and because counties with higher food insecurity and lower education consistently show poorer diet quality, including lower fiber intake, higher added sugar intake, and worse overall Healthy Eating Index scores [41,61,62]. Furthermore, although we could not directly account for food consumption patterns, national studies confirm that rural populations tend to consume ultra-processed diets, with less fiber and more added sugar, which could contribute to higher obesity prevalence independent of herbicide exposure [63,64]. Therefore, our adjustment of selected dietary proxies help reduce confounding by diet, which may be systematically poorer in rural areas, although cannot fully address the lack of direct county-level measures of diet and physical activity. Other exposures common in agricultural settings, including the use of other types of pesticides (e.g., insecticides, rodenticides, etc.), air pollution, and heavy metals, may also confound or interact with herbicide–obesity associations, and their omission represents an additional limitation.

### 4.3. New Dimensions in Exposure Assessment

Our study contributes methodologically by leveraging publicly available county-level herbicide application data to approximate population exposures. This ecological approach enabled us to probe potential exposure–response relationships on a national scale. We observed, for example, that counties with the highest herbicide application levels tended to have some of the highest obesity prevalence, with obesity rates plateauing around ~31% in the most agriculturally intensive areas. Although based on observational correlations, this pattern raises the hypothesis of threshold effects whereby metabolic health risks may accelerate once herbicide use (and by inference, community exposure) exceeds a certain level. Our analysis, which captured geographic variability and multiple chemicals simultaneously, provides a more comprehensive real-world exposure scenario than single-chemical studies. By accounting for dozens of U.S. states and a spectrum of herbicide compounds, we capture the complex environmental conditions under which human populations actually live. Furthermore, our ecological approach adds a new dimension to exposure science by marrying large-scale data with mixture modeling to yield insights that are not apparent when studying one chemical or one location at a time. This integrated exposure assessment is a significant step forward in bridging the gap between epidemiological observations and mechanistic toxicology.

### 4.4. Strengths, Limitations, and Future Directions

Strengths of our study include that we analyzed a large, nationally representative dataset covering six years (2013–2018) and over 3000 counties, which provides ample statistical power and broad generalizability. We integrated high-quality data from federal sources—including USGS agricultural herbicide use estimates and CDC-modeled county obesity prevalence—and adjusted for a range of demographic and socioeconomic covariates to reduce confounding. To address missing data, we employed multiple imputation methods customized to the dataset, maximizing the use of available information and limiting bias from incomplete records. Notably, we applied a rigorous mixture modeling approach (quantile g-computation) to estimate the joint effect of 13 herbicides on obesity. This method, developed specifically for epidemiologic mixtures, allowed us to calculate an overall effect estimate (Psi) for the herbicide blend while yielding weights for each component. The quantile-based approach is robust to distributional extremes and multicollinearity, enhancing our confidence that the observed mixture effect is not an artifact of one highly prevalent chemical. Taken together, the study design and analytical techniques provide a robust triangulation of evidence at the population level, complementing prior individual-level study.

Our study has several limitations. First, the study is ecologic and analysis was performed on data that is aggregated at county level, thus results cannot be interpreted as causal effects at the individual level, and indeed, spatiotemporal aggregation may obscure finer-scale relationships. However, a gold-standard randomized controlled trial (RCT) for these exposures and outcomes would establish causality at an individual level, but would be unethical. And, while a longitudinal study would strengthen causality, one of this size would be prohibitively expensive and, if observational, would not establish causality on its own. Thus, while it has stated limitations, our study leverages publicly available datasets to explore associations between a ubiquitous set of exposures and a chronic health outcome on national scales. Our study is based on an assumption that county-level herbicide application is correlated with local human exposure, and results may be influenced by unmeasured county-level factors such as diet, physical activity, or other correlated exposures. We address this by including county-level random effects, adjusting for rurality, and controlling for multiple socio-demographic variables. Aggregation at the county level may introduce a modifiable area unit problem. Our herbicide use metric is a surrogate for human exposure and does not account for individual behaviors or chemical drift/dynamics, which may lead to non-differential exposure misclassification and bias estimates toward the null. County obesity prevalence was obtained from a modeling method (BRFSS small-area estimates) that has its own uncertainty. However, by including year as a fixed effect and county random effects we attempted to account for differences associated with time and spatially associated variability. Fourth, while quantile g-computation is a powerful tool for mixtures, it computes additive effects of increasing all exposures by one quantile and de facto assumes linearity unless nonlinear terms are explicitly incorporated into a model. We found minimal evidence of pairwise interactions among herbicides in supplementary analyses, suggesting the additivity assumption was reasonable. Still, very high correlation between certain herbicides (e.g., metolachlor and metolachlor-S) required us to drop one variable to avoid collinearity, highlighting a general challenge in multi-pollutant studies. Our current dataset (county-level CDC obesity data with additional county-level covariates from the ACS) comprises population rates by county and thus does not allow stratification by sex, and does not include occupational proportions. Therefore, we were unable to stratify by sex or by rural occupational status, other than including rate of unemployment by county. Future studies with individual-level data could address these important sources of heterogeneity.

Finally, our focus was on obesity as outcome; we did not examine other metabolic outcomes (such as diabetes, hypercholesterolemia, or hypertension rates) in this analysis. It remains possible that herbicide mixtures impact various metabolic health indicators differently, an aspect a future study will explore. In view of these limitations, our study should be considered exploratory.

We recommend several directions for further research. Controlled longitudinal studies—for example, following birth cohorts or agricultural communities over time—are needed to test the temporality of herbicide exposure and obesity onset, which our ecologic design cannot establish. Incorporating individual-level exposure data (e.g., urinary or blood biomarkers of herbicides) would greatly strengthen causal inference by reducing exposure misclassification and allowing dose–response relationship assessment. Indeed, one recent longitudinal study of young adults (the CHAMACOS cohort) reported that cumulative glyphosate exposure was associated with elevated metabolic syndrome risk, illustrating the value of detailed exposure tracking [51]. Future studies might also consider experimental and mechanistic investigations, such as animal or cellular models of herbicide mixtures, to unravel how these chemicals might jointly disrupt metabolic regulation. For instance, do low-dose combinations of glyphosate, 2,4-D, and atrazine induce greater adipogenesis or insulin resistance in vivo than each compound alone? Questions like this remain unanswered, and toxicological research could elucidate potential synergistic or antagonistic interactions at the molecular level. Additionally, examining spatial patterns using finer geographic resolutions (e.g., census tract data, as in recent environmental determinant studies) could help identify localized “hotspots” of metabolic disease tied to herbicide use, thereby refining intervention targets.

## 5. Conclusions

In summary, this ecological analysis adds to our understanding of environmental influences on metabolic health. While it cannot prove causation, the alignment of our population-level findings with evidence from individual-based studies [5,15,51,52] strengthens inference that chronic herbicide exposures may be contributing to chronic obesity. Our study offers a broad spatiotemporal perspective on this issue, suggesting that areas of heavy herbicide application are associated with obesity hotspots in the community. This big-picture view complements mechanistic and epidemiologic research at the micro level, and underscores the importance of considering real-world chemical mixtures in public health analyses. Ultimately, tackling complex problems like obesity requires integrating information across scales—from molecules and individuals up to communities and ecosystems. By highlighting an environmental dimension to obesity that operates at the county scale, our findings aim to stimulate more nuanced investigations and preventive strategies. Caution is warranted in interpretation due to our study design, but the evidence presented here contributes to a growing consensus that environmental chemical exposures are an important piece of the metabolic health puzzle.

## Figures and Tables

**Table 1:** NCHS Urban-rural classification scheme (2013).

Category	Category Description
Large Central Metro	NCHS-defined “central” counties, MSAs 1 million+ population
Large Fringe Metro	NCHS-defined MSA fringe areas, 1 million+ population
Medium Metro	Counties within MSAs of 250,000 to 999,999
Small Metro	Counties within MSAs of 50,000 to 249,999
“Micropolitan”	Counties in NCHS-defined Micropolitan statistical areas
Non-core	Counties not in Micropolitan statistical areas

(https://www.cdc.gov/nchs/data-analysis-tools/urban-rural.html, accessed on 1 March 2025).

**Table 2:** Population/demographic variables (N = 18,382), years 2013–2018. Summarized from imputed data.

Covariate (%)		Mean	Median	IQR
**Outcome**	Obese	30.9	31.0	[29.0, 34.0]
**Risk factors**	Smoking	19.4	19.0	[16.0, 22.0]
	Uninsured	17.0	16.9	[12.0, 21.0]
	Unemployed	6.7	6.3	[4.7, 8.1]
	Rurality	60.3	60.7	[35.2, 88.9]
	Food-Insecure	13.6	14.0	[11.0, 16.0]
**Education**	High School Graduate	84.1	86.0	[79.0, 91.0]
	Some College	55.8	55.8	[47.6, 64.3]
**Age**	Population < 18 years	22.7	22.6	[20.7, 24.4]
	Population > 64 years	17.4	17.1	[11.5, 13.8]
**Race (percent)**	Non-Hispanic White	77.9	86.3	[68.2, 93.9]
	Black/African American	8.8	2.0	[0.6, 9.3]
	Asian	1.3	0.6	[0.4, 1.1]
	Hispanic	8.9	3.5	[1.9, 8.0]
	Native American	1.9	0.6	[0.3, 1.1]
	Pacific Islander	0.08	0.0	[0.0, 0.10]
	**Level**		**Number Counties**	**(Percent)**
^1^ **NCHS Urban-Rural Designation**	Large Central Metro		351	(1.9%)
	Large Fringe Metro		2130	(11.6)
	Medium Metropolitan		2190	(11.9)
	Small Metro		2094	(11.4)
	Micropolitan		3803	(20.7)
	Non-core		7814	(42.5)

^1^ https://www.cdc.gov/nchs/data-analysis-tools/urban-rural.html.

**Table 3:** Estimates (per standard deviation exposure) from adjusted **single-pollutant** mixed-effects models for associations between herbicide application rates per hectare and county-level obesity rates. Results are shown as forest plot visualizations in Figure 1.

Herbicide	Estimate	95% Confidence Interval	*p*-Value	FDR Adjusted
acetochlor	0.23	0.17, 0.30	<0.0001	<0.0001
atrazine	0.16	0.095, 0.23	<0.0001	<0.0001
glufosinate	0.08	0.038, 0.12	<0.0001	<0.0001
glyphosate	0.29	0.21, 0.36	<0.0001	<0.0001
metolachlor	0.12	0.08, 0.16	<0.0001	<0.0001
metolachlor + metolachlor-S	0.24	0.18, 0.29	<0.0001	<0.0001
metolachlor-S	0.18	0.13, 0.23	<0.0001	<0.0001
pendimethalin	0.11	0.046, 0.16	<0.0001	<0.0001
herbicide 2-4D	0.09	0.036, 0.135	0.001	0.002
dimenthenamid-P	0.05	0.01, 0.097	0.02	0.04
dicamba	−0.013	−0.05, 0.024	0.49	0.62
chlorothanonil	−0.02	−0.078, 0.049	0.66	0.72
paraquat	−0.01	−0.0, 0.038	0.67	0.72
trifluralin	−0.008	−0.077, 0.06	0.81	0.81

**Table 4:** Mixture effects of county-level herbicide applications on obesity rates from crude and adjusted quantile g-computation mixture models [46]. Results for crude (mixture and FIPS code only) and adjusted models, including interactants with nonzero EIM identified via interaction forest runs, i.e., glyphosate × dicamba, metolachlor × paraquat, and metolachlor × acetochlor, are shown below the solid line. Estimated Psi (ψ) values represent increases in county-level obesity rates per quantile increase in county-level ex-posure mixture.

Model	Coefficient	Estimate	95% Confidence Interval	*p*-Value
Crude	intercept	29.5	29.4, 29.6	<0.0001
	Psi (ψ)	**1.0**	0.94, 1.1	<0.0001
Adjusted	intercept	28.0	27.6, 28.4	<0.0001
	Psi (ψ)	**0.71**	0.65, 0.76	<0.0001
Crude + interactants	intercept	29.4	29.3, 29.6	<0.0001
	Psi (ψ)	**1.4**	1.2, 1.6	<0.0001
Adjusted + interactants	intercept	28.1	27.7, 28.5	<0.0001
	Psi (ψ)	**0.68**	0.50, 0.86	<0.0001

## Data Availability

Herbicide and pesticide data are available for download from the USGS Pesticide national synthesis project e-pest site e-pest site by county and year. See https://water.usgs.gov/nawqa/pnsp/usage/maps/county-level/ (accessed on 15 December 2023). County-level outcome and covariate summaries are freely available from the CDC, and for ease of access are also aggregated and offered by county at the County Health Rankings project of the University of Wisconsin Population Health Institute: https://www.countyhealthrankings.org.

## Abbreviations

The following abbreviations are used in this manuscript:

ACS	American Community Survey
BKMR	Bayesian Kernel Machine Regression
BRFSS	Behavioral Risk Factor Surveillance System
CDC	Centers for Disease Control
CHAMACOS	a Spanish slang term referring to children or young people
EIM	Effect Importance Measure
FIPS	Federal Information Processing Standards
NCHS	National Center for Health Statistics
OMB	Office of Management and Budget
PUR	Pesticide Use Reporting
USGS	United States Geological Survey

## Appendix A

**Imputing missing data.**

Most missing units were in the county-level pesticide application data with missingness at most 20% (Dimenthenamid-P) and at minimum 0.06% (Glyphosate). For demographic data, smoking rates and rate of high school graduation had 7.6% and 12.5% missing units, respectively. All other demographic data, and the outcome variable (county-level obesity rate), were complete.

Before creating our imputation model, the dataset was visualized using heatmaps to check for structure or patterns within the missing units. All missing values were multiply imputed using a fully conditional chained equations approach implemented in R via the mice package. This method imputes each variable with its own imputation model and covariates, which are specified with a custom block diagonal prediction matrix.

In our imputation model, demographic variables were grouped and imputed together using predictive mean matching. Herbicide data were grouped together with the complete urban–rural indicator variable since rurality is strongly associated with application levels (Table A2) and imputed using a random forest method. We created 10 imputed datasets, each given 20 iterations to converge. All analysis was performed on and results pooled over ten imputed datasets using Rubin’s Rules [42].

**Additional figures and tables.**

**Table A1:** Herbicides applied, kilograms per (hectare) ^1^, by county.

Herbicide	Min	Q25	Median	Q75	Max
trifluralin	0	47.5	290.2	1063.1	118,984.0
pendimethalin	0	152.1	705.6	1940.6	127,641.6
paraquat	0	95.7	424.9	1418.0	87,700.0
metolachlor-S	0	264.1	2049.6	11,095.6	193,956.5
metolachlor + metolachlor-S	0	365.8	2727.5	13,709.0	202,600.2
metolachlor	0	86.4	608.8	2280.8	70,608.4
herbicide 2-4D	0	802.9	2884.3	8000.0	199,793.4
glyphosate	0.1	2015.7	15,359.3	65,956.5	594,336.0
glufosinate	0	29.8	277.9	1456.4	87,516.1
dimenthenamid-P	0	47.8	429.2	1807.8	497,78.35
dicamba	0	91.4	369.1	1318.4	112,221.9
chlorothanonil	0	21.8	127.5	661.7	176,201.4
atrazine	0	312.4	2300.6	15,193.0	743,835.8
acetochlor	0	139.2	1335.5	9354.0	153,269.7

^1^ Exposures by year from Epest county-level estimates 2013–2018. Pooled over 10 imputed datasets.

**Table A2:** Estimates from adjusted **single-pollutant** mixed-effects models for associations between herbicide application rates per hectare and county-level obesity rates. Regressions are on exposures quantized by quintile; reference level is first quintile (Q1). First quintile (Q1) is the lowest 20%.

Herbicide	Quantile	Estimate	95% CI	*p*-Value
herbicide 2-4D	Q2	0.18	0.067, 0.296	0.002
	Q3	0.37	0.24, 0.51	<0.0001
	Q4	0.46	0.32, 0.61	<0.0001
	Q5	0.57	0.40, 0.73	<0.0001
acetochlor	Q2	0.17	0.05, 0.29	0.007
	Q3	0.42	0.27, 0.57	<0.0001
	Q4	0.58	0.40, 0.76	<0.0001
	Q5	0.67	0.47, 0.87	<0.0001
atrazine	Q2	0.18	0.07, 0.30	0.002
	Q3	0.34	0.20, 0.48	<0.0001
	Q4	0.60	0.44, 0.76	<0.0001
	Q5	0.93	0.74, 1.13	<0.0001
chlorothanonil	Q2	−0.07	−0.17, 0.04	0.20
	Q3	−0.16	−0.3, −0.05	0.007
	Q4	−0.18	−0.3 −0.06	0.004
	Q5	−0.09	−0.25, 0.06	0.24
dicamba	Q2	0.24	0.13, 0.34	<0.0001
	Q3	0.31	0.19, 0.43	<0.0001
	Q4	0.46	0.32, 0.58	<0.0001
	Q5	0.53	0.38, 0.67	<0.0001
dimenthenamid-P	Q2	0.052	−0.05, 0.15	0.3
	Q3	0.15	0.03, 0.28	0.02
	Q4	0.28	0.14, 0.42	<0.0001
	Q5	0.29	0.14, 0.45	<0.0001
glufosinate	Q2	0.13	0.024, 0.23	0.016
	Q3	0.23	0.10, 0.36	<0.0001
	Q4	0.39	0.25, 0.53	<0.0001
	Q5	0.54	0.37, 0.70	<0.0001
glyphosate	Q2	0.14	0.02, 0.3	0.03
	Q3	0.38	0.22, 0.6	<0.0001
	Q4	0.82	0.64, 1.0	<0.0001
	Q5	1.0	0.8, 1.2	<0.0001
metolachlor	Q2	0.19	0.08, 0.30	0.001
	Q3	0.38	0.25, 0.5	<0.0001
	Q4	0.56	0.41, 0.7	<0.0001
	Q5	0.76	0.60, 0.93	<0.0001
metolachlor + metolachlor-S	Q2	0.18	0.05, 0.30	0.006
	Q3	0.38	0.23, 0.54	<0.0001
	Q4	0.70	0.54, 0.89	<0.0001
	Q5	0.95	0.75, 1.14	<0.0001
metolachlor-S	Q2	0.18	0.06, 0.30	0.004
	Q3	0.32	0.18, 0.46	<0.0001
	Q4	0.63	0.47, 0.79	<0.0001
	Q5	0.83	0.65, 1.0	<0.0001
paraquat	Q2	0.018	−0.09, 0.13	0.7
	Q3	0.087	−0.04, 0.21	0.16
	Q4	0.19	0.06, 0.32	0.005
	Q5	0.22	0.07, 0.37	0.004
pendimethalin	Q2	−0.02	−0.13, 0.10	0.8
	Q3	0.049	−0.08, 0.18	0.5
	Q4	0.19	0.04, 0.33	0.011
	Q5	0.23	0.08, 0.39	0.003
trifluralin	Q2	0.03	−0.08, 0.14	0.6
	Q3	0.21	0.10, 0.33	0.001
	Q4	0.20	0.09, 0.33	0.004
	Q5	0.15	−0.01, 0.3	0.06

**Table A3:** Mixture effects of spatially distributed herbicides on Obesity rates by county from crude and adjusted quantile g-computation mixture models, stratified by NCHS Urban-Rural designation. Crude models are mixture and FIPS only (included as fixed effect in crude and adjusted models). Estimated Psi (ψ) values represent increase in obesity per quantile increase in mixture exposure. A quantile division of 15 for the mixture was selected by maximizing Z-scores and checking underlying model fits.

NCHS Designation	Model	Coefficient	Estimate	95% CI	*p*-Value
Large Central Metro	Crude	intercept	25.4	24.4, 26.5	<0.0001
		Psi (ψ)	0.57	−0.14, 1.3	0.11
	Adjusted	intercept	25.8	24.4, 27.3	<0.0001
		Psi (ψ)	0.77	0.42, 1.12	<0.0001
Large Fringe Metro	Crude	intercept	27.8	27.4, 28.2	<0.0001
		Psi (ψ)	1.23	0.96, 1.50	<0.0001
	Adjusted	intercept	27.8	27.2, 28.4	<0.0001
		Psi (ψ)	0.59	0.4, 0.77	<0.0001
Medium Metro	Crude	intercept	29.5	29.1, 29.9	<0.0001
		Psi (ψ)	0.71	0.47, 0.95	<0.0001
	Adjusted	intercept	28.8	28.3, 29.4	<0.0001
		Psi (ψ)	0.73	0.55, 0.9	<0.0001
Small Metro	Crude	intercept	29.5	29.1, 29.9	<0.0001
		Psi (ψ)	0.92	0.67, 1.2	<0.0001
	Adjusted	intercept	29.5	28.9, 30.1	<0.0001
		Psi (ψ)	0.70	0.50, 0.90	<0.0001
Micropolitan	Crude	intercept	29.4	29.1, 29.7	<0.0001
		Psi (ψ)	1.38	1.2, 1.6	<0.0001
	Adjusted	intercept	30.0	29.5, 30.4	<0.0001
		Psi (ψ)	0.88	0.72, 1.04	<0.0001
Non-Core	Crude	intercept	30.6	30.35, 30.8	<0.0001
		Psi (ψ)	0.65	0.5, 0.8	<0.0001
	Adjusted	intercept	30.4	30.1, 30.7	<0.0001
		Psi (ψ)	0.47	0.35, 0.59	<0.0001

**Table A4:** Rurality, obesity rates, and annual herbicide application per hectare for top 14 herbicides by NCHS Urban-Rural designation (2013 standard). Highest applications across rural–urban sectors are shown in boldface dark red, and second highest in lighter red.

Feature	^1^ NCHS Urban-Rural Designation					
	Large Central Metro	Large Fringe Metro	Medium Metro	Small Metro	Micropolitan	Non-core
Rurality (%)	2.4	40.3	40.5	46.5	52.3	79.1
Obesity rate (%)	26.2	29.5	30.5	30.8	31.3	31.5
Median Herbicide Application (kg/hectare)						
acetochlor	143	580	440	**1290**	**1374**	959
atrazine	321	1617	1241	**2611**	**2734**	1858
chlorothanonil	170	168	**184**	**172**	134	95
dicamba	53	201	267	411	**428**	**444**
dimenthenamid-P	61	134	113	293	**356**	**309**
glufosinate	36	154	135	**286**	**301**	220
glyphosate	2686	8449	7538	**20,220**	**18,851**	16,838
herbicide 2-4D	656	1858	2338	3073	**3404**	**3316**
metolachlor	73	351	336	552	**662**	480
metolachlor + metolachlor-S	494	1808	1554	2800	**3590**	2411
metolachlor-S	421	1352	1139	2251	**2581**	1769
paraquat	91	310	376	**509**	**456**	412
pendimethalin	136	441	587	**902**	**856**	728
trifluralin	53	102	96	221	**277**	**292**

^1^ https://www.cdc.gov/nchs/data-analysis-tools/urban-rural.html.
